# Fine-tuning RNA extraction protocols for polysaccharide-rich freshwater red algae (Rhodophyta)

**DOI:** 10.17912/micropub.biology.001180

**Published:** 2024-04-10

**Authors:** Sunil Tiwari, Morgan L Vis

**Affiliations:** 1 Arizona State University, Tempe, Arizona, United States; 2 Ohio University, Athens, Ohio, United States

## Abstract

Freshwater red algae are important primary producers, widely distributed, contributing significantly to nutrient cycling in aquatic ecosystems. Recent studies have focused on identifying the effect of different environmental conditions such as light on the gene expression in photosynthesis pathways. However, obtaining the necessary RNA quantity and quality for sequencing from these algae has been challenging. Although RNA extractions have been optimized for model organisms, RNA extraction for non-model organisms, such as gelatinous (polysaccharide-rich) red algae, requires considerable troubleshooting. The common freshwater red alga, *Batrachospermum gelatinosum* , was used to test protocols. The extraction efficiency of various sample disruption methods in combination with seven RNA extraction kits was compared. Using a 2-minute disruption procedure with a modification of TRIzol ^™^ Plus RNA Purification Kit (PureLink ^™^ RNA Mini Kit + Trizol ^™^ ) protocol resulted in significantly higher RIN scores (p < 0.05) and high RNA concentration, compared to other methods. The fine-tuned protocol yielded quality RNA (RIN>7) in high concentrations for subsequent sequencing.

**Figure 1.**
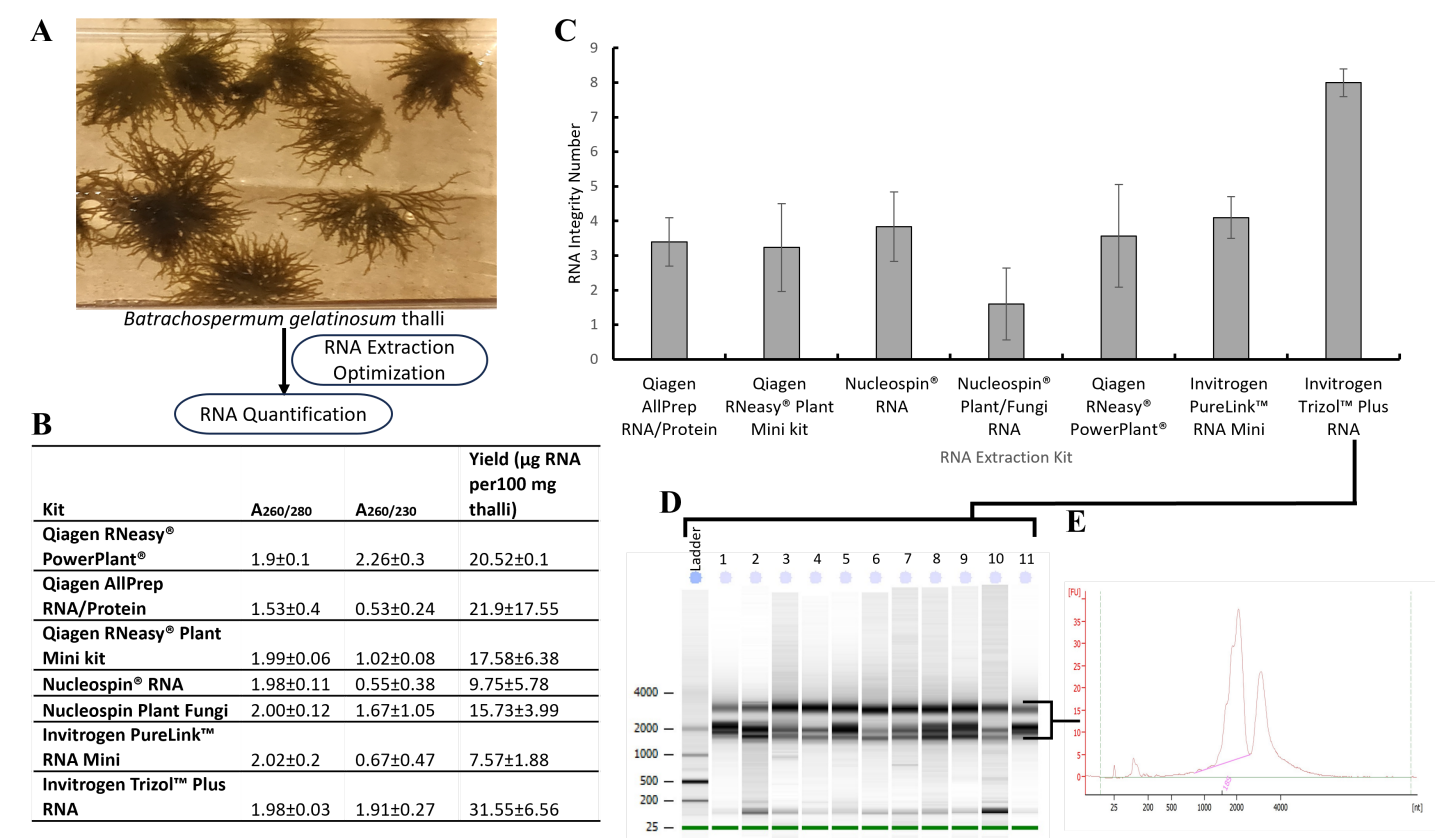
(A) RNA extraction workflow: *Batrachospermum gelatinosum* thalli were extracted in triplicate using numerous protocols. (B) NanoDrop measurement of RNA extracts (260/280 nm, 260/230 nm ratios, and RNA yields). (C) Mean (n=3) RNA integrity number (RIN) of RNA extracted for seven kits. (D) Gel image generated by the Bioanalyzer of RNA extracted using fine-tuned protocol. Dominant bands of 18S and 28S RNA are visible in all preparations. (E) A representative Bioanalyzer electropherogram from sample 11 in Figure D.

## Description

With the advent of RNA sequencing technologies, numerous RNA extraction kits are now available. However, these extraction kits were developed primarily for model organisms and there is a need for optimization of these kits for RNA extraction from non-model organisms (Johnson et al., 2012; Sasi et al., 2023; Singh et al., 2024) . The red alga, *Batrachospermum* , is exclusively freshwater and an important constituent of these systems as a primary producer (Qiu et al., 2023) . The presence of phycobilisome antenna proteins in red algae differentiates them from plants and green algae. Due to the presence of a phycobilisome antenna protein complex, these red algae have unique light regulation mechanisms (Gantt et al., 2003) . Several recent studies have focused on demonstrating the effect of different environmental conditions on these algae at a molecular level with transcriptomics being one of the important tools for differential gene expression (Nan et al., 2018; Evans & Vis, 2020) . However, extracting high-quality RNA from gelatinous freshwater red algae, like *B. gelatinosum* , can be challenging.

These red algae are polysaccharide-rich and often polysaccharides interfere with RNA extraction by co-precipitating with nucleic acids, leading to contamination of the RNA (Mundt et al., 2018) . Another issue is that the complex polysaccharides can hinder the disruption of cells during the lysis step and exoenzymes rapidly degrade RNA once cells are disrupted making the immediate stabilization of RNA challenging (Sasi et al., 2023; Jensen et al., 2023) . In addition, secondary metabolites, such as tannins and polyphenols, may inhibit RNA extraction enzymes and degrade RNA or may co-purify with RNA during extraction (Carpinetti et al., 2021) . Standard RNA extraction kits are not specifically optimized for these unique characteristics of gelatinous algae, thus we set out to test a suite of available kits to determine the efficiency and optimization of the extraction process to obtain RNA quality and quantity for sequencing.

We used seven RNA extraction kits with *B. gelatinosum* (Figure A). Each disruption/extraction protocol (see Methods below) was performed with three replicates. To evaluate the final protocol, twelve additional extractions were performed. The RNA yield is provided as µg of RNA per 100 mg of algal biomass (µg/100 mg thalli).

With the Qiagen AllPrep RNA/Protein extraction kit, the RNA quality and quantity were low, with a yield of 21.9±17.55 µg (Figure B) and mean RIN scores of 3.4±0.4 (Figure C). To ensure that the issue was specific to *B. gelatinosum* , RNA was extracted (in triplicate) from 100 mg Arabidopsis tissue using the same methods. Good RNA quality and quantity were obtained (RIN: 8±0.8, Yield: 113.5±21.6 µg).

One of the major challenges working with polysaccharide-rich algae is achieving proper tissue disruption to ensure both high RNA yield and quality. We implemented several tissue disruption methods with the Qiagen RNeasy® Plant Mini kit. The initial 2-minute bead-beating protocol (2 cycles of 1 minute each with 30 sec gaps between cycles at 3000 rpm) was insufficient for cell disruption. This determination was evident from visual inspection and RNA yield (7.2±1.4 µg) as well as the RIN score (0.9±0.8). A 3-minute protocol resulted in potential overheating; even though the RNA quantity increased (43.3±9.3 µg) the RIN scores did not improve (2.5±0.5). Subsequently, we adopted an innovative approach to pre-freezing the tubes with beads at -80 °C for eight hours before RNA extraction. This modification led to a minor increase in RIN score (3.2±0.7) and even though an increased cell disruption was observed, the yield of 17.58±6.38 µg was still low (Figure B, C). In addition, we tried mortar pestle grinding, which improved RNA yield (31.3±5.1 µg), but RIN scores (2.9±1.8) were similar to those from bead beating. Since the algal sample is gelatinous, even frozen, it did not pulverize easily, and grinding for a longer period led to the thawing of the sample and probably more degradation of RNA. Despite initially deeming the 2-minute protocol insufficient, we observed that pre-freezing the tubes with glass beads at -20 °C or -80 °C for eight hours led to improved cell disruption, and thus we adopted this modified approach for all subsequent extractions.

RNA extracted from both Nucleospin® RNA and Nucleospin® Plant/Fungi RNA did not increase the RNA yield (9.75±5.78 and 15.73±4 µg, respectively) and RIN scores (3.8±0.6 and 1.2±1.2, respectively) were still low (Figure B, C). RNeasy® Powerplant kit resulted in a higher RNA yield (20.52±0.1 µg), compared to Nucleospin® kits but the RIN score (3.6±0.9) did not increase. Given that all extractions conducted with -80 °C frozen samples were not yielding the needed results, fresh samples were extracted using the RNeasy® Powerplant kit. With the fresh samples, the RNA yield (91.3±13.4 µg) improved considerably, but the RIN (3.5±0.9) scores remained low (Data not presented in the figure).

Next, we investigated the Invitrogen PureLink ^™^ RNA Mini Kit and Invitrogen TRIzol ^™^ Plus RNA Purification Kit. For these RNA extractions, the tubes and beads were kept at -20 ^ᵒ^ C for 8 hours before bead beating, and 50 mg of sample was used. With the PureLink ^™^ RNA Mini Kit, slightly higher RIN scores (4.4±0.6) were obtained, but no improvement in RNA yield (7.57±1.88 µg) was observed (Figure B, C). We tested TRIzol ^™^ Plus RNA Purification Kit, which showed similar RNA yield (20.3±1.5 µg) and RIN scores (3.8±0.6) (Data not presented in the figure). However, the addition of 10 µL of β-ME (2-Mercaptoethanol) per 1mL of Trizol ^™^ , as per the troubleshooting section of the manual, resulted in significantly higher RIN scores (8±0.2) compared to other kits (p < 0.05) and adequate yield (31.55±6.56 µg) (Figure B, C). To confirm the reproducibility of the protocol, we performed 12 additional extractions (Figure D), which resulted in the RIN scores in the range of 5.8-9.3 (mean: 7.4±1.1) and mean yield of 37.6±1.6 µg. The gel image for these extracts showed bright bands of 28S and 18S RNA genes (Figure D). A representative Bioanalyzer electropherogram confirmed minimal degradation of RNA (Figure E). For this preparation, the use of prechilled beads and tubes likely helped maintain a low temperature during thalli disruption avoiding excessive heat generation and thus preventing RNA degradation. Similarly, maintaining the low temperature during thalli disruption was crucial to slowing enzymatic activity and preserving the integrity of RNA molecules. In addition, the presence of β-ME in the homogenate could have prevented polyphenol oxidation in addition to eliminating the RNases released during cell disruption, providing an added layer of protection for RNA integrity throughout the extraction process. In summary, sample disruption was done with the 2-minute protocol (2 cycles of 1 minute each with 30-sec gaps between cycles) at 3000 rpm, tubes along with beads were stored at -20 ^ᵒ^ C for 8 hours before bead beating, 10 µL of β-ME per 1 mL of Trizol ^™^ was added and absolute ethanol (100%) was used for washing the columns. On-column DNase treatment was done following the on-column PureLink ^™^ DNase treatment protocol.

To conclude, our analysis of various disruption methods and RNA extraction kits revealed challenges associated with isolating RNA from gelatinous freshwater red algae. Different RNA extraction methods yielded varying results in terms of the RNA concentration and quality (RIN). TRIzol™ Plus RNA Purification Kit, with specific modifications, consistently yielded high-quality RNA, demonstrating its efficacy in handling the challenging properties of *B. gelatinosum* . Notably, the Trizol™ Plus RNA kit showed a significant difference in RIN scores compared to the other protocols consisting of various disruption methods and kits; there was no significant difference in RNA concentration (p > 0.05) between these kits. The importance of RNA quality and its impact on the reliability of downstream processing such as RNA sequencing, and qPCR reactions cannot be overemphasized. The pre-chilling of beads/tubes, disruption time, and modified TRIzol™ Plus RNA Purification Kit with β-ME is a protocol that works consistently for *B. gelatinosum* and is likely suitable for a variety of freshwater as well as marine algae especially red algae making it possible to obtain high-quality RNA extraction for downstream applications such as RNA sequencing.

## Methods

Macroscopic gametophyte thalli of *Batrachospermum gelatinosum* were collected from Monday Creek at Carbon Hill, Ohio, USA (39°30'02.5"N 82°14'47.8" W). In the laboratory, the algal specimens were washed with distilled water to remove epiphytes and other visible contaminants. The algae were blotted on a tissue and approximately 100 mg was placed in a 1.5 mL tube, flash-frozen in liquid nitrogen, and stored at -80°C until RNA extraction. Triplicate extractions were conducted for each kit or modified protocol. All RNA extractions were performed using ~100 mg of tissue (unless noted otherwise). Homogenization of algal tissue was completed in Omni Bead Ruptor 12 Homogenizer. For all the RNA preps, the final elution volume was 50 µL RNase-free water.

RNA extraction was conducted using the Qiagen AllPrep RNA/Protein extraction kit (Catalog no. 80404) following the standard protocol. For cell lysis, APL buffer was added to the algal tissue and disrupted using 3 mm beads (2 cycles each of 45 seconds with a speed of 3000 rpm) in Omni Bead Ruptor 12 Homogenizer. The RNA was eluted using 50 µL RNase-free water.

A set of extractions was conducted using the Qiagen RNeasy® Plant Mini kit (Catalog no. 74904) following manufacturer protocol. Samples were disrupted using 3 mm beads and 450 µl Buffer RLT in an Omni bead beater at 3000 rpm. The bead-beading time was tested to study the effect on RNA quality and quantity. The following homogenization protocols were tested: 2-minute (2 cycles of 1 min each with 30 sec between cycles) and 3-minute (2 cycles of 1.5 min each with 30 sec between cycles) with 3 mm glass beads. In addition, as a potential improvement to the protocol, grinding in liquid N _2_ using mortar and pestle replaced cell disruption with a bead beater. The mortar and pestle were washed in ultrapure water, wrapped in aluminium foil, and double autoclaved. Samples were ground in liquid-N _2,_ and the powder was immediately transferred to a 1.5 µL tube and 450 µL RLT buffer was added.

Two sets of extractions were conducted using Nucleospin® RNA and Nucleospin® Plant/Fungi RNA kit standard protocols. The sample was ground in liquid nitrogen using mortar and pestle as recommended by the protocol and the homogenized sample was immediately transferred to a 1.5 µL tube. Then, 350 µL RA1 buffer and 3.5 µL β-ME (2-Mercaptoethanol) were quickly added.

RNA was extracted using the RNeasy® PowerPlant® kit following the standard protocol. Sample disruption was completed with the 2-minute protocol at 3000 rpm. In addition to extracting RNA from frozen samples, fresh samples were tested with this kit.

Two sets of extractions were conducted using PureLink ^™^ RNA Mini Kit and TRIzol ^™^ Plus RNA Purification Kit (PureLink ^™^ RNA Mini Kit + Trizol ^™^ ) following the standard protocol. Sample disruption was completed with a 2-minute protocol at 3000 rpm. The tubes used for bead beating were stored at -20 ^O^ C for 8 hours before bead beating. For the sample disruption, 10 µL of β-ME per 1mL of Trizol ^™^ was added and the extraction was conducted following the standard protocols.

**Statistical Analysis:**

Welch's analysis of variance (ANOVA) was used to identify significant differences among data sets. This analysis was conducted using the R-studio package with R version 4.0.2 (R Development Core Team, 2008) . The Tukey's Honestly Significant Difference (HSD) test was used for pairwise comparisons between the various protocols utilized.

## References

[R1] Carpinetti PA, Fioresi VS, Ignez da Cruz T, de Almeida FAN, Canal D, Ferreira A, Ferreira MFDS (2021). Efficient method for isolation of high-quality RNA from Psidium guajava L. tissues.. PLoS One.

[R2] Evans JR, Vis ML (2020). Relative expression analysis of light-harvesting genes in the freshwater alga Lympha mucosa (Batrachospermales, Rhodophyta).. J Phycol.

[R3] Gantt Elisabeth, Grabowski Beatrice, Cunningham Francis X. (2003). Antenna Systems of Red Algae: Phycobilisomes with Photosystem ll and Chlorophyll Complexes with Photosystem I. Light-Harvesting Antennas in Photosynthesis.

[R4] Jensen Timo, Saleh Livia, Bents Dominik, Krohn Steffen, Wu Yu-Chen, Mucke Maria, Boje Ammelie Svea, Veltel Stefan, Hennig Steffen, Piker Levent, Peipp Matthias, Labes Antje (2023). Optimised protocols for RNA extraction from a broad taxonomic range of algae. Journal of Applied Phycology.

[R5] Johnson MT, Carpenter EJ, Tian Z, Bruskiewich R, Burris JN, Carrigan CT, Chase MW, Clarke ND, Covshoff S, Depamphilis CW, Edger PP, Goh F, Graham S, Greiner S, Hibberd JM, Jordon-Thaden I, Kutchan TM, Leebens-Mack J, Melkonian M, Miles N, Myburg H, Patterson J, Pires JC, Ralph P, Rolf M, Sage RF, Soltis D, Soltis P, Stevenson D, Stewart CN Jr, Surek B, Thomsen CJ, Villarreal JC, Wu X, Zhang Y, Deyholos MK, Wong GK (2012). Evaluating methods for isolating total RNA and predicting the success of sequencing phylogenetically diverse plant transcriptomes.. PLoS One.

[R6] Mundt Florian, Heinrich Sandra, Hanelt Dieter (2018). RNA isolation from taxonomically diverse photosynthetic protists. Limnology and Oceanography: Methods.

[R7] Nan F, Feng J, Lv J, Liu Q, Xie S (2018). Transcriptome analysis of the typical freshwater rhodophytes Sheathia arcuata grown under different light intensities.. PLoS One.

[R8] Qiu Mingyu, Wang Fei, Nan Fangru, Feng Jia, Lü Junping, Liu Qi, Liu Xudong, Xie Shulian (2023). Geographical distribution of Batrachospermaceae genera in Asia and its environmental factors. Journal of Oceanology and Limnology.

[R9] R Core Team, R. (2013). R: A language and environment for statistical computing. http://www.R-project.org

[R10] Sasi Shina, Krishnan Saranya, Kodackattumannil Preshobha, Shamisi Aysha AL, Aldarmaki Maitha, Lekshmi Geetha, Kottackal Martin, Amiri Khaled M. A. (2023). DNA-free high-quality RNA extraction from 39 difficult-to-extract plant species (representing seasonal tissues and tissue types) of 32 families, and its validation for downstream molecular applications. Plant Methods.

[R11] Singh Vivek Vikram, Naseer Aisha, Sellamuthu Gothandapani, Jakuš Rastislav (2024). An Optimized and Cost-Effective RNA Extraction Method for Secondary Metabolite-Enriched Tissues of Norway Spruce (Picea abies). Plants.

